# On reliable discovery of molecular signatures

**DOI:** 10.1186/1471-2105-10-38

**Published:** 2009-01-29

**Authors:** Roland Nilsson, Johan Björkegren, Jesper Tegnér

**Affiliations:** 1 Computational Biology, Department of Physics, Linköping University, SE58183 Linköping, Sweden; 2 Unit of Computational Medicine, King Gustav V Research Institute, Department of Medicine, Karolinska Institutet, SE17176 Stockholm, Sweden

## Abstract

**Background:**

Molecular signatures are sets of genes, proteins, genetic variants or other variables that can be used as markers for a particular phenotype. Reliable signature discovery methods could yield valuable insight into cell biology and mechanisms of human disease. However, it is currently not clear how to control error rates such as the false discovery rate (FDR) in signature discovery. Moreover, signatures for cancer gene expression have been shown to be unstable, that is, difficult to replicate in independent studies, casting doubts on their reliability.

**Results:**

We demonstrate that with modern prediction methods, signatures that yield accurate predictions may still have a high FDR. Further, we show that even signatures with low FDR may fail to replicate in independent studies due to limited statistical power. Thus, neither stability nor predictive accuracy are relevant when FDR control is the primary goal. We therefore develop a general statistical hypothesis testing framework that for the first time provides FDR control for signature discovery. Our method is demonstrated to be correct in simulation studies. When applied to five cancer data sets, the method was able to discover molecular signatures with 5% FDR in three cases, while two data sets yielded no significant findings.

**Conclusion:**

Our approach enables reliable discovery of molecular signatures from genome-wide data with current sample sizes. The statistical framework developed herein is potentially applicable to a wide range of prediction problems in bioinformatics.

## Background

**Figure 1:**
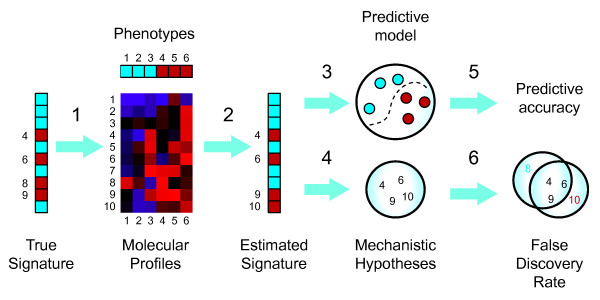
**Signature discovery**. Molecular signatures (1) are markers for a particular cell or tissue phenotype. Signatures are discovered from a given set of molecular profiles (e.g., gene expression profiles) together with phenotype labels (2). Signatures have dual uses, both as predictive models (3) and for discovery of molecular mechanisms (4). While it is well-known how to assess predictive accuracy (5), the method proposed herein is the first to control signature FDR (6), enabling reliably discovery.

Molecular signatures are sets of genes, mRNA transcripts, proteins, genetic variants or other variables that can be used as markers for a particular cell or tissue phenotype, such as a cancerous or diabetic state. Signatures have a two-fold purpose: they may be useful for disease diagnosis or risk assessment (*prediction*), but they may also implicate molecules not previously known to be involved in the underlying molecular pathology (*discovery*), as illustrated in Figure 1A. Signature discovery differs from simple correlation or differential expression testing in that molecular signatures may account for multivariate effects and consists only of the variables most directly correlated with given phenotype. The signature approach has been especially popular for cancer diagnosis based on gene expression profiling, and several studies have proposed signatures for specific cancer types [1-5]. A prominent example is the breast cancer signature discovered by van't Veer *et al*. [4], which is currently being validated in a clinical trial [6].

Unfortunately, existing computational approaches often fail to distinguish between the different objectives of prediction and discovery. If molecular signatures are to be used for discovery, then the primary objective is to control the false discovery rate (FDR) with respect to the optimal (true) signature. On the other hand, if the end goal is an accurate predictor, then the FDR of the gene signature is not important in itself. However, it has hitherto not been possible to directly address FDR control, since an operational definition of the optimal signature (a "gold standard") has not been available. Therefore, current methods for signature discovery resort to optimizing prediction accuracy, implicitly assuming that the FDR is thereby kept reasonably low, even though there is no *a priori *reason to assume that this is the case. Recently, the *stability *of a signature, that is, the expected overlap between signatures derived from replicated experiments, has been suggested as an alternative quality measure [7,8]. Signatures derived from cancer gene expression data have been found to be unstable, raising concerns that existing signature discovery methods may not be sound [9,10]. While the stability measure seems intuitively reasonable and cleverly avoids the gold standard problem, it has not been shown that low stability actually indicates high FDR.

In this paper, we build upon a recently discovered operational definition of the optimal signature to study the actual FDR in signature discovery. First, we demonstrate that high FDR can occur even with very accurate predictors. Therefore, current methods for signature discovery that focus on optimizing prediction accuracy offer no means of controlling the FDR. Second, we show that signatures can be highly unstable even when the FDR is kept low. Thus, reliable signature discovery may be possible in spite of the recent reports of unstable signatures in cancer [9,10]. Third, we propose a novel hypothesis testing procedure based on our definition of the optimal signature that for the first time directly addresses signature FDR. We show that our method achieves FDR control on simulated data. Application to well-known cancer data sets uncovers three novel molecular signatures for leukemia, colon and breast cancer.

## Results

### The optimal signature

For simplicity, we will consider a two-class prediction setting throughout, although the methods could be generalized to other prediction problems as well. A *predictor *is then a function *g *:X↦Y, where we take X = ℝ^*n *^and Y = {-1, +1}. The *accuracy *of a predictor *g *is 1 minus the probability of error or *risk R*(*g*) = *P*(*g*(*X*) ≠ *Y*). An optimal predictor, denoted g* is one with maximal accuracy. An optimal signature can be defined as a minimal set of variables S* such that the optimal predictor obtained using only these variables is at least as accurate as any predictor obtained with any other set, that is,

(1) $$\forall S:\forall g_{S}:R(g_{S^{*}}^{*})\leq R(g_{S}),$$

where *gS *denotes a predictor on the subspace X_*S *_of X corresponding to the variable set *S*. Unfortunately, this criterion does not yield a unique S* in general, and there are examples of data distributions such that no tractable (polynomial-time) algorithms exist for computing S* [[11], pp. 562]. Consequently, most research has focused on heuristic algorithms for discovering approximate signatures with near-optimal prediction accuracy [12].

While this approach has been largely successful at attaining good predictive accuracy, the lack of a "gold standard" has rendered direct evaluation of error rates for signature discovery algorithms impossible. To address this problem, we have recently shown [13] that using a mild restriction on the class of data distributions, the set S* becomes unique and can be expressed as

(2) $$S^{*}=\{i:R(g_{\left\{1,...,i-1,i+1,...,n\right\}}^{*})>R(g_{\left\{1,...,n\right\}}^{*})\}.$$

That is, S* consists precisely of the variables *i *such that the error probability of the optimal predictor g* increases when *i *is removed. The required restriction is that the data density *f *(*x*) is everywhere strictly positive. This condition is satisfied by nearly all commonly used statistical models, including the exponential family, and we believe that it is reasonable for biological data. A formal proof of the correctness of (2) is given in Additional File 1.

Note that S* may be quite different from the set of variables that are marginally correlated with the phenotype (*e.g*., differentially expressed genes). This is because some correlated variables may be "redundant" for prediction: while these do contain information about the phenotype, that information is also present in other variables, so that the redundant variables are excluded from S*. Indeed, it can be proved that S* only contains variables *X*_*i *_that are conditionally dependent on *Y *regardless of what other variable set is conditioned on [13]. In this sense, S* constitutes the variables "directly" correlated with *Y*. Moreover, some variables may be predictive only when considered together with certain other variables in a multivariate fashion, and thus S* may contain variables that are not detectable by standard univariate methods [14].

The simple form (2) immediately suggests a general, linear-time, asymptotically correct algorithm for discovering S* from data, as described elsewhere [13]. Here, we make use of the fact that (2) permits S* to be calculated for any given data distribution, thus providing the gold standard required for evaluating signature discovery methods and developing hypothesis testing procedures.

### Accurate predictions despite high signature FDR

**Figure 2:**
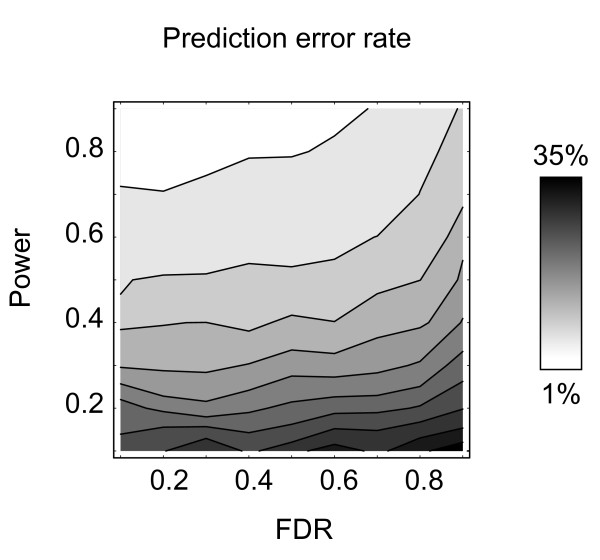
**Good predictive accuracy despite high FDR**. Probability of prediction error for the Support Vector Machine (gray level) as a function of signature false discovery rate (FDR) and statistical power (fraction of true positives). Nearly horizontal level curves indicate weak dependence on FDR.

First, we tested whether high prediction accuracy implies a low false discovery rate with respect to S*. We performed a simulation study on a simple two-class prediction problem using a multivariate normal distribution with *n *= 1, 000 variables, of which 10% were in S* (see Methods for details). In each run, a signature *S *was chosen to achieve a given power and FDR with respect to S*, whereafter a Support Vector Machine (SVM) classifier was trained on a sample from the corresponding subspace of the data distribution. We found that FDR as high as 50% did not degrade predictive accuracy discernably, provided that statistical power was sufficient (Figure 2). Thus, prediction accuracy is not a valid measure of the reliability of a signature in terms of false positives.

The likely reason for this behavior is that modern predictive methods such as the SVM have internal mechanisms for suppressing noise (regularization). They are therefore rather insensitive to false positives within the signature. For prediction purposes, it is more important that the signature does contain some true positives genes, while a large fraction of irrelevant genes may be tolerated without degrading predictive accuracy. As a consequence, discovering signatures by optimizing prediction accuracy should not be expected control FDR, as we will further demonstrate below.

### Unstable Signatures with Low FDR

**Figure 3:**
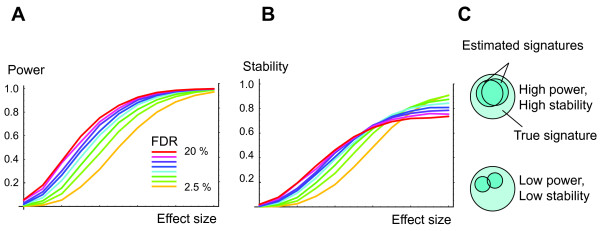
**Signatures with low FDR may be unstable**. Left, statistical power *vs*. effect size (arbitrary units) for varying FDR. Middle, stability, defined as the average normalized overlap between two signatures *vs*. effect size and FDR. Right, illustration of how power affects stability.

To investigate the relation between signature stability and FDR, we conducted a second simulation experiment, again with *n *= 1, 000 variables. Here, each variable was conditionally independent of all others within each class, so that S* has the form

$$S^{*}=\{i:E[x_{i}|Y=+1]\neq E[X_{i}|Y=-1]\},$$

and can be discovered by simply testing the marginal distributions for a nonzero mean difference. For this we used Student's t-test with the Benjamini-Hochberg correction for FDR control, since the t-test has optimal power in this case and the FDR can be controlled exactly [15]. Nevertheless, we found that the resulting signatures can be very unstable (Figure 3). For small effect sizes where power was low, stability was also low, despite a stringent FDR. Conversely, with strong effects and high power, stability was high, even with a high FDR. Also, the dependence of stability on FDR was different between low- and high-power regimes, indicating that the relationship between these measures is complicated and data-dependent. Clearly, unstable signatures need not contain many false positives. In the low power regime, the situation is rather that small signatures are being selected more or less at random from a large set of true positives, resulting in small overlap between experiments (Figure 3). Hence, in situations where many genes are weakly associated with a given phenotype and power is limited, it is simply not feasible to reproduce molecular signatures in independent experiments, even with the most stringent and correct methods. This implies that the lack of reproducibility observed for cancer gene expression signatures [7,8] is not necessarily problematic. The same mechanism may also account for the low reproducibility of whole-genome association studies of complex diseases [16], where many genes are believed to be weakly associated with a given disease trait.

### A Statistical Framework for Signature Discovery

The above results show that neither predictive accuracy nor stability are relevant measures of signature FDR. To directly control false discovery rates for signature discovery, we instead propose a general method for directly testing the hypothesis *i *∈ S* for each variable. From equation (2) it follows that a generally applicable test statistic is

$$T_{i}=\hat{R}(g_{\left\{1,...,i-1,i+1,...,n\right\}}^{*})-\hat{R}(g_{\left\{1,...,n\right\}}^{*}),$$

where $\hat{R}$ is an estimated error probability, for example a cross-validated error estimate. This statistic is asymptotically correct for any data distribution, that is, with a sufficiently large sample size, the globally optimal solution will always be found [13]. However, the sample sizes required for reasonable performance could be very large, since the error rate estimate $\hat{R}$ is uncertain. For particular types of predictors, it is therefore preferable to develop specialized statistics. As we are interested in applications to gene expression data, where simple prediction rules tend to work well, we here consider linear classifiers of the form *g*(*x*) = sign (∑_*i*_*w*_*i*_*x*_*i*_). It is easy to see that in this case, equation (2) reduces to

$$S^{*}=\{i:w_{i}^{*}\neq 0\},$$

where $w_{i}^{*}$ denote the weights of the optimal classifier. Assuming that the classifier used is consistent, we have that $w_{i}^{*}$[*w*_*i*_] → $w_{i}^{*}$ as sample size increases. Hence, in this case we can equivalently test the null hypothesis E[*w*_*i*_] = 0. More complicated parametric forms such as polynomials in *x*_*i *_could be used in a similar way, although the number of weights would increase accordingly.

Since the statistical distribution of *w*_*i *_is unknown, we used a bootstrap technique to test whether E[*w*_*i*_] = 0. By sampling with replacement from the given data set and re-training the classifier on each sample, we obtain *B *vectors $w_{1}^{*},...,w_{B}^{*}$. For each variable *i*, the corresponding $w_{1i}^{*},...w_{Bi}^{*}$ are then used to obtain a bootstrap confidence interval for *w*_*i*_. This interval is inverted to obtain a bootstrap *p*-values *p*_*i *_for each variable *i *(that is, the null hypothesis is rejected at level *α *if the (1 - *α*) confidence interval does not cover zero). Importantly, this procedure preserves the full dependency structure of the data distribution. Finally, FDR control was performed using the Benjamini-Hochberg procedure [15].

### Simulation Experiments

**Figure 4:**
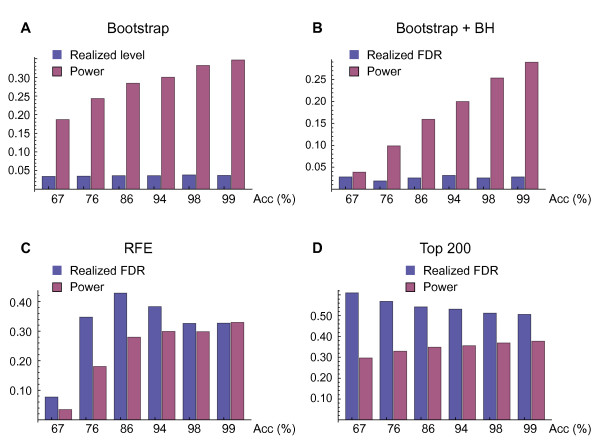
**Controlling error rates for gene signatures**. **A: **Realized level and power for the bootstrap test at 5% nominal level. **B: **Realized FDR, power and stability for signatures selected by the bootstrap test after Benjamini-Hochberg (BH) correction. Here the nominal FDR was set at 5%. **C: **Same as (B) for signatures selected by recursive feature elimination (RFE). **D: **Same as (B) for signatures selected as the top 200 genes. Acc, classifier accuracy.

To validate our method, we conducted simulations using two-class data with 1, 000 variables and 100 samples. To model the variable dependencies often present in gene expression data, we used a class-conditional multivariate Gaussian distribution with precision matrices generated randomly as previously described [17]. For this distribution class, it is straightforward to calculate S* (see methods). We chose sampling parameters so that S* constituted approx. 200 variables on average (since S* depends on the randomly chosen covariance matrix, its size fluctuates somewhat). We evaluated three linear classification methods: the Support Vector Machine (SVM) [18], the Kernel Fisher Discriminant (KFD) [19] and the Weighted Voting (WV) algorithm of Golub *et al*. [2]. Since the results were highly similar for all of these, we here only present results for the SVM (see Additional File 2 for KFD and VW). For each learning method and across a range of effect sizes, our bootstrap test produced correct *p*-values, while power increased with increasing effect size (Figure 4A). This demonstrates that the bootstrap test is sound. After correcting for multiplicity using the procedure of Benjamini and Hochberg [15], we verified that our method did control FDR at nominal levels (Figure 4B). Power was limited however, especially for predictors with low accuracy. We therefore expect that for high-dimensional data, predictors must be quite accurate in order to yield reliable signatures. We also verified that the power of our bootstrap method approaches 1 as sample size increases, as one would expect (see Additional File 2). However, power depends on a number of distribution properties, and it is difficult to make predictions about the sample sizes required in practise from simulations.

We repeated the simulation study using the popular Recursive Feature Elimination (RFE) method [20] to discover signatures. While this method did produce accurate predictive models (data not shown), we observed that FDR was high (above 40% in this experiment) and depended on the effect size in an unpredictable manner. Indeed, optimizing prediction accuracy by RFE does not guarantee a reliable signature. High FDR was also observed when choosing the signature *S *as a fixed-size "top list" by the rank according to the *w*_*g *_statistics (Figure 4D). We have also previously observed high FDR for other methods that optimize the signature for prediction accuracy [21]. Often, these methods attempt to include more variables in the signature when the prediction problem is harder, thus sacrificing FDR control for better predictive accuracy. Conversely, for less difficult prediction problems, many true positives may be removed from the signature because they do not influence predictive power discernably.

### Application to Cancer Gene Expression

We applied our method together with the SVM prediction method to analyze a number of publicly available cancer gene expression data sets (Table 1). For the data sets by van't Veer [4] and Wang [5] where the SVM had poor accuracy, the bootstrap method did not call any genes significant. Note that these signatures may still be useful for prediction; the fact that no genes are called significant merely demonstrates that it is not possible to ascertain which genes are responsible for the predictive accuracy. For the remaining data sets, we found that higher predictive accuracy tends to result in greater power, in accordance with our simulation results. The largest signature, obtained for the data set by Golub *et al*. [2], contained over 500 genes at 5% FDR (see Additional Files 3, 4 and 5 for complete gene lists).

**Table 1:** Results on cancer gene expression data

Data set (ref.)	*n*	MCF,%	CV,%	TA,%(ref.)	BS	BS_0_	RFE	RFE_0_	DE
Golub (2)	72	32	97.0 ± 4.2	99.3 (28)	537	0	35	154	1007
Singh (4)	136	43	92.6 ± 3.0	81.1 (27)	99	0	48	312	3807
Alon (1)	62	35	81 ± 7.2	97.9 (29)	19	0	55	94	303
Wang (6)	286	37	65 ± 4.3	N/A	0	0	261	1250	106
van't Veer (5)	97	47	62 ± 8.4	N/A	0	0	42	153	1

Results are ordered by prediction accuracy. *n*, number of samples; MCF, minority class frequency; CVA, balanced cross-validated prediction accuracy, mean ± std.dev.; TA, balanced prediction accuracy of bootstrap signature on an independent test set (reference given in parentheses); BS, significant genes using the bootstrap with SVM at 5% FDR; RFE, genes chosen by recursive feature elimination; BS_0 _and RFE_0_, gene chosen by the bootstrap and RFE methods respectively on randomized data. DE, differentially expressed genes using the t-test at 5% FDR.

As a negative control, we applied our bootstrap test on randomized versions of each original data set where the phenotype values were randomly permuted, corresponding to the complete null hypothesis. This yielded zero significant genes in each case, confirming that we do not obtain spurious findings. In contrast, when applying the RFE method to randomized data, we consistently obtained even larger signatures than with the real data sets. We also tested each signature on an independent data set, confirming that the signatures are indeed predictive.

For comparison, we performed a conventional differential expression test for each data set using the t-test statistic with the Benjamini-Hochberg correction (Table 1). This identified a substantially larger set of genes than the bootstrap method – in one case, more than half of the genes tested were significant. This illustrates the ability of the gene signature approach to distinguish the genes directly related to the phenotype variable from a much larger set of differentially expressed genes: many of the latter turn out to be "redundant" for prediction, meaning that they are correlated with the phenotype only indirectly, through genes in S*.

## Discussion

Molecular signatures offer a systematic way to focus on the genes most directly associated with a given phenotype, and may yield valuable insights into the underlying biological system. It is therefore unfortunate that the reliability of signatures *per se *is poorly understood. Since no gold standard for signature discovery has been available, validation of discovered signatures often amounts to mining the scientific literature for documented connections between the phenotype being studied and the elements (genes) of a hypothesized signature. However, this approach is necessarily biased and rather speculative: it is by no means clear that a gene should be included in a predictive signature simply because it is somehow "related" to the phenotype. For example, approximately 25% of all known human genes have some documented relation to cancer [14], but it is unlikely that all of these should be included in an optimal signature for cancer prediction.

To address this issue, we have herein developed a statistical method for signature discovery based on a formal definition of the "gold standard" optimal signature. This allows for assessing the reliability of signatures without detailed knowledge of the biological system. To our knowledge, our method is the first to provide statistical guarantees for the reliability of molecular signatures, although we note that random forests are similar to our bootstrap testing scheme and also give indications of what variables are important for prediction.

For two of the gene expression data sets investigated, including the well-studied cancer data by van't Veer *et al*. [4], our method did not call any genes significant, indicating that these data sets did not contain sufficient information to uncover gene signatures at the specified false discovery rate (5%). We emphasize that this does not necessarily mean that it is infeasible to construct predictive models for these studies, but merely that it is difficult to determine which genes are responsible for the predictive accuracy. In this sense, discovering reliable gene signatures can be a harder problem than obtaining accurate predictors. Prediction and signature discovery are two separate problems, and must be treated differently.

For simplicity, we have here restricted our analysis to two-class problems and linear predictors. However, the proposed method is applicable to any learning method for which a reasonably well-powered statistic can be derived to test the signature null hypothesis. Continuous phenotype variables can easily be addressed by substituting the classification methods used herein for regression methods, such as ridge regression [22] or the relevance vector machine [23]. General methods for handling non-linear dependencies have also been described [13,24], although it is unclear whether signature discovery from gene expression data would benefit from these more complex models with currently available sample sizes.

Some technical issues remain to be considered. First, testing the null hypothesis E[*w*_*i*_] = 0 is technically correct only in the limit of large samples where E[*w*_*i*_] → $w_{i}^{*}$. While our simulation studies indicate correct behavior for the sample sizes tested, this issue warrants further study. Second, bootstrap hypothesis testing is known to provide only approximate *p*-values, satisfying the inequality *P*(*p *≤ *α*) ≤ *α *+ O(1/*l*), where *l *is the sample size [25]. While the additional term O(1/*l*) was negligible in our simulations, this should be verified in each particular case before applying bootstrap testing. A possible future improvement could be to estimate this term from simulations and correct the bootstrap *p*-values accordingly, thereby "calibrating" the method.

## Conclusion

As we have shown, neither predictive accuracy nor stability constitute valid measures of FDR for molecular signatures. Indeed, highly accurate predictions may be obtained despite an FDR as high as 50% (Figure 2), while in situations where many weak effects are present and statistical power is low, signatures can be unstable at an FDR as low as 2.5% (Figure 3). This analysis explains at least some of the difficulties with reproducing cancer gene expression signatures [7,8] and possibly also the similar reproducibility problems of recent association studies in complex diseases [16]. Moreover, it suggests that this lack of reproducibility need not be problematic.

We have developed and validated a statistical hypothesis testing framework that for the first time provides false discovery rates control for signature discovery. In application to cancer gene expression, we have showed that reliable signature discovery is feasible with currently available sample sizes. Many important problems in bioinformatics are prediction problems and may benefit from reliable signature discovery. We therefore hope that our method will be of general interest.

## Methods

Signature stability is defined as the normalized expected overlap between two signatures *S, S' *derived from independent, replicate experimental data sets,

(3) $$\text{Stability}=E_{S,S^{\prime }}\left[\frac{\left|S\cap S^{\prime }\right|}{\max\{\left|S\cup S^{\prime }\right|,1\}}\right],$$

where E denotes statistical expectation. The stability is always between 0 (no expected overlap) and 1 (complete overlap).

Simulations were performed with data drawn from two-class multivariate Gaussian distributions *f *(*x *| *y*) = *N *(*yμ*, Σ) with equal class frequencies, covariance matrix Σ independent of the class (phenotype) variable *y *and varying degrees of class separation to achieve different effect sizes. Results were averaged over 100 randomly selected Gaussian distributions. for each parameter setting tested. We measure the effect size of the resulting prediction problem by the expected SVM accuracy. Here the accuracy was computed exactly for each SVM from the data density: for any given *μ *and Σ, a separating hyperplane with normal vector *w *has classification accuracy

$$\text{Acc}(w)=\frac{1}{2}\left[1+\text{erf}\left(\frac{w^{T}\mu }{\sqrt{2w^{T}\Sigma w}}\right)\right],$$

where $\text{erf}(x)=2\pi^{-1/2}\int_{0}^{x}e^{-t^{2}}dt$ is the error function.

To evaluate signature error rates, we used the fact that for *f *(*x *| *y*) = *N *(*yμ*, Σ), the optimal separating hyperplane has normal vector w* = Σ^-1 ^*μ*, and so the optimal set S* can be determined as the nonzero components $w_{i}^{*}$ of this vector.

For hypothesis testing, we used a parametric bootstrap with *B *= 50 repetitions, fitting a Gaussian distribution *N *(*μ*_*i*_, *σ*_*i*_) to the observed $w_{1g}^{*},...w_{Bg}^{*}$ prior to computing two-sided *p*-values. In preliminary studies, the difference between this method and a nonparametric bootstrap with *B *= 1000 was negligible, while the parametric version is computationally more efficient since a much smaller *B *can be used. The SVM [18], KFD [19] and VW [2] methods were implemented as previously described. In all experiments, the SVM *C*-parameter and the KFD regularization parameter were set to 1. Recursive Feature Elimination (RFE) was performed as previously described [20], using the radius-margin bound [26] as accuracy measure and removing 20% of the genes in each iteration.

Microarray data sets [1-5] were preprocessed by removing genes displaying small variation, keeping the 5,000 most variable genes in each case, except for the data sets by van't Veer *et al*. [4] and Alon *et al*. [1] which were preprocessed in a similar fashion by the original authors. Genes were normalized to zero mean and unit standard deviation prior to SVM training, following standard practise for kernel methods. Independent test data sets [27-29] were normalized in the same fashion. No other preprocessing was done prior to classifier training or testing.

Since many data sets were had low minor class frequencies are (Table 1), performance was evaluated with the balanced accuracy measure

$${\text{Acc}}_{\text{balanced}}=\frac{{\text{Acc}}_{+}+{\text{Acc}}_{-}}{2},$$

where Acc_+ _and Acc_- _are the accuracy measures for each class. Except for the independent test sets, these were measured by cross-validation, where in each round a randomized set consisting of 2/3 of the samples was used for training, and the remaining 1/3 was used for testing. Splits were balanced so that class frequencies were equal between training/test data. Mean and standard deviation of the balanced test error over 50 cross-validation repetitions are reported.

## Authors' contributions

RN, JB and JT designed research; RN performed research; RN and JT wrote the paper.

## Supplementary Material

Additional file 1 **Proofs.** This document provides proofs of uniqueness and optimality of the optimal signature S*. Click here for file

Additional file 2 **KFD and WV methods, and convergence with increasing sample size.** This figure shows the results corresponding to Figure 4 for the Kernel Fisher Discriminant (A-B) and Weighted Voting classification methods (C-D). Also shown is the convergence of the bootstrap method for the SVM classifier (E), where power approaches 1 as sample size increases. Click here for file

Additional file 3 **Gene signature for the Alon data set.** Excel file detailing the gene signature discovered by the bootstrap method using the SVM classifier. The corresponding signature from Recursive Features elimination is also provided for reference. Click here for file

Additional file 4 **Gene signature for the Golub data set.** Excel file detailing the gene signature discovered by the bootstrap method using the SVM classifier. The corresponding signature from Recursive Features elimination is also provided for reference. Click here for file

Additional file 5 **Gene signature for the Singh data set. **Excel file detailing the gene signature discovered by the bootstrap method using the SVM classifier. The corresponding signature from Recursive Features elimination is also provided for reference. Click here for file
