# Supplementary material for: On reliable discovery of molecular signatures
Source: BMC Bioinformatics. 2009 Jan 29;10:38. doi: 10.1186/1471-2105-10-38 (PMC2646701; doi:10.1186/1471-2105-10-38)
Supplement: Additional file 1 — Proofs. This document provides proofs of uniqueness and optimality of the optimal signature S*. [file 1471-2105-10-38-S1.pdf]

ON RELIABLE DISCOVERY OF MOLECULAR SIGNATURES ADDITIONAL FILE 1: OPTIMALITY AND UNIQUENESS OF S^*

The notation here is the same as in the paper. Sets are used as indexes X_S to denote $(X_i)_{i \in S}$. The complement of a set S is denoted $\neg S$, and we write $X_{\neg i}$ for all elements of X except X_i. Probability density functions (over the continuous $\mathcal{X}$) are denoted f, while or probability mass functions (over the discrete $\mathcal{Y}$) are denoted p.

We first state our definition of S^*.

Definition 1. *For any set $S \subseteq \{1, \ldots, n\}$, let g^*_S denote the optimal classifier over the corresponding domain $\mathcal{X}_S$. The optimal signature is defined as*

$$S^* = \{i : R(g^*_{\neg i}) > R(g^*)\},$$

where for any classifier g we define $R(g) = P(g(X) \neq Y)$.

The following establishes uniqueness of the optimal classifier g^*, and thereby uniqueness of S^*.

Theorem 2. *For any $f(x, y)$ such that $P(p(y \mid X) = 1/2) = 0$, the optimal classifier is unique, that is,*

$$P(g(X) \neq g^*(X)) > 0 \iff R(g) > R(g^*)$$

for all classifiers g.

Proof. Take any classifier g and fix an $y \in \mathcal{Y}$. We have that

$$\begin{aligned} P(g(x) \neq Y) - P(g^*(x) \neq Y) &= (2p(y \mid x) - 1)(1_{\{g^*(x)=y\}} - 1_{\{g(x)=y\}}) \\ &= |2p(y \mid x) - 1| 1_{\{g^*(x) \neq g(x)\}}. \end{aligned}$$

Integrating with respect to $f(x)dx$ we obtain

$$R(g) - R(g^*) = \int_{\mathcal{X}} |2p(y \mid x) - 1| 1_{\{g^*(x) \neq g(x)\}} f(x) dx.$$

The assumption $P(p(y \mid X) = 1/2) = 0$ is equivalent to $|2p(y \mid x) - 1| > 0$ with probability 1. Therefore, the integral is positive if and only if

$$\int_{\mathcal{X}} 1_{\{g^*(x) \neq g(x)\}} f(x) dx = P(g(X) \neq g^*(X)) > 0.$$

□

The following lemma establishes an equivalent formulation of S^* which is useful in proving its optimality.

Lemma 3. *For any $f(x, y)$ such that $P(p(y \mid X) = 1/2) = 0$, it holds that*

$$S^* = \{i : P\left(g^*(X) \neq g^*_{\neg i}(X_{\neg i})\right) > 0.\}$$

Proof. Follows immediately the definition and Lemma 2. □

We now prove that $g^*_{S^*}$ is optimal for strictly positive distributions.

Theorem 4. *For any $f(x, y)$ satisfying $f(x) > 0$ and $P(p(y \,|\, X) = 1/2) = 0$, the set S^* satisfies $g^*_{S^*}(X_{S^*}) = g^*(X)$ with probability 1.*

Proof. Take any $i, j \notin S^*$. By Lemma 3, $g^*(X) = g^*_{\neg i}(X_{\neg i}) = g^*_{\neg j}(X_{\neg j})$ with probability 1. We will show this implies that $g^*(X)$ is constant with respect to $X_{i,j}$ with probability 1. To see this, fix any x and assume to the contrary that there exists a point

$$x' = (x_{\neg\{i,j\}}, x'_{i,j})$$

such that $g^*(x) \neq g^*(x')$. Then we can construct a third point

$$x'' = (x_{\neg\{i,j\}}, x_i, x'_j),$$

and by the assumptions this point must satisfy both

$$g^*(x'') = g^*_{\neg i}(x''_{\neg i}) = g^*_{\neg i}(x'_{\neg i}) = g^*(x')$$

and

$$g^*(x'') = g^*_{\neg j}(x''_{\neg j}) = g^*_{\neg j}(x_{\neg j}) = g^*(x).$$

Since $f(x'') > 0$, this is a contradiction. Therefore we must have

$$g^*(X) = g^*_{\neg\{i,j\}}(X_{\neg\{i,j\}})$$

with probability 1. Repeating this argument for each element of $\neg S^*$ yields the result. □
