# Supplementary material for: On reliable discovery of molecular signatures
Source: BMC Bioinformatics. 2009 Jan 29;10:38. doi: 10.1186/1471-2105-10-38 (PMC2646701; doi:10.1186/1471-2105-10-38)

Additional File 2

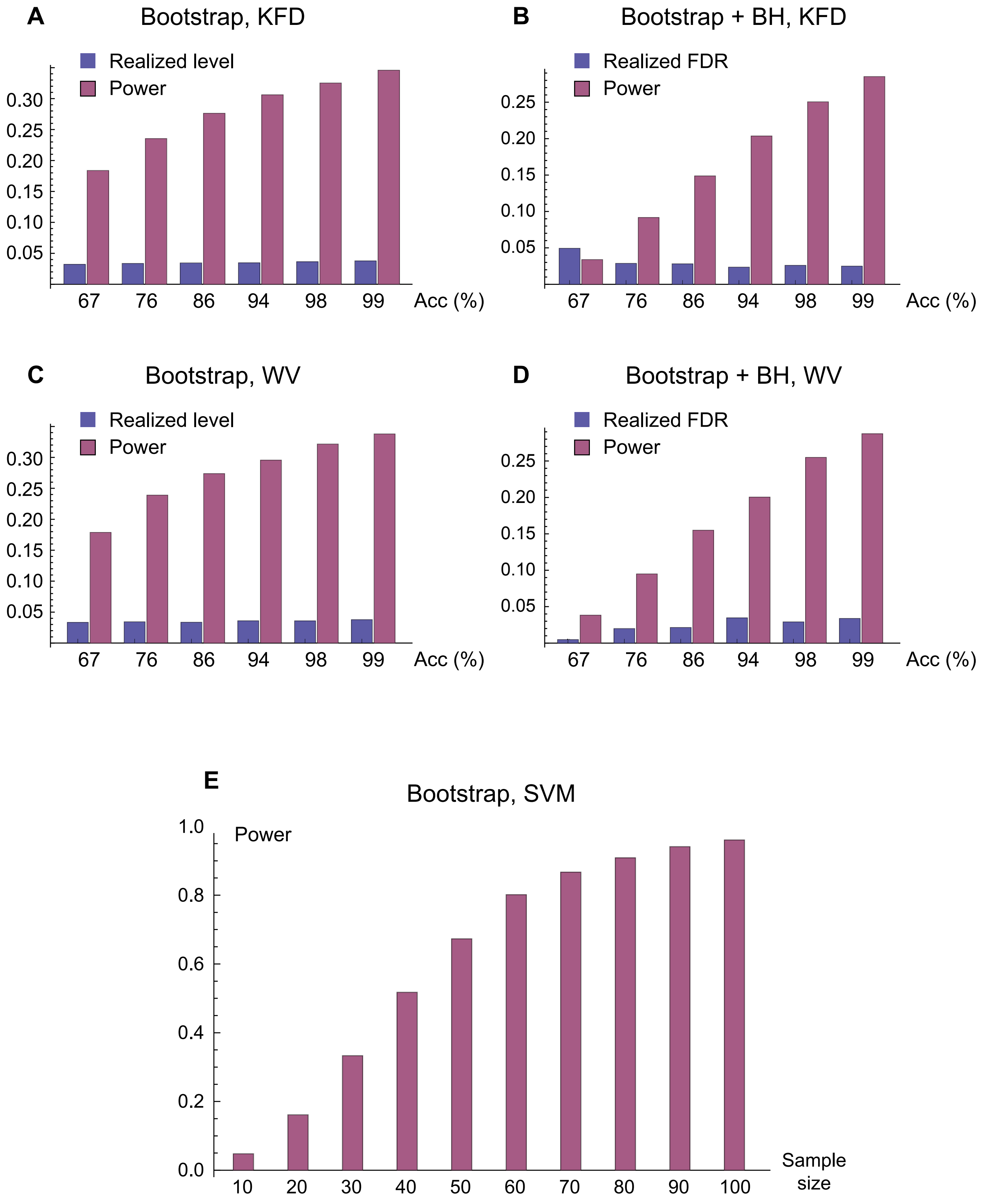

Supplement: Additional file 2 — KFD and WV methods, and convergence with increasing sample size. This figure shows the results corresponding to Figure 4 for the Kernel Fisher Discriminant (A-B) and Weighted Voting classification methods (C-D). Also shown is the convergence of the bootstrap method for the SVM classifier (E), where power approaches 1 as sample size increases. [file 1471-2105-10-38-S2.pdf]
